# Theoretical prediction and atomic kinetic Monte Carlo simulations of void superlattice self-organization under irradiation

**DOI:** 10.1038/s41598-018-24754-9

**Published:** 2018-04-26

**Authors:** Yipeng Gao, Yongfeng Zhang, Daniel Schwen, Chao Jiang, Cheng Sun, Jian Gan, Xian-Ming Bai

**Affiliations:** 1 Fuels Modeling and Simulation, Idaho National Laboratory (INL), Idaho Falls, ID 83415 USA; 2 Advanced Characterization Department, Idaho National Laboratory (INL), Idaho Falls, ID 83415 USA; 3 Department of Materials Science and Engineering, Virginia Polytechnic Institute and State University, 460 Old Turner Street Blacksburg, VA, 24061 USA

## Abstract

Nano-structured superlattices may have novel physical properties and irradiation is a powerful mean to drive their self-organization. However, the formation mechanism of superlattice under irradiation is still open for debate. Here we use atomic kinetic Monte Carlo simulations in conjunction with a theoretical analysis to understand and predict the self-organization of nano-void superlattices under irradiation, which have been observed in various types of materials for more than 40 years but yet to be well understood. The superlattice is found to be a result of spontaneous precipitation of voids from the matrix, a process similar to phase separation in regular solid solution, with the symmetry dictated by anisotropic materials properties such as one-dimensional interstitial atom diffusion. This discovery challenges the widely accepted empirical rule of the coherency between the superlattice and host matrix crystal lattice. The atomic scale perspective has enabled a new theoretical analysis to successfully predict the superlattice parameters, which are in good agreement with independent experiments. The theory developed in this work can provide guidelines for designing target experiments to tailor desired microstructure under irradiation. It may also be generalized for situations beyond irradiation, such as spontaneous phase separation with reaction.

## Introduction

Nanoscale self-organization has led to the formation of a variety of two-dimensional (2D) and three-dimensional (3D) patterned structures such as nanoparticle superlattice^1,2^, surface quantum dots and ripples^3,4^, nanodroplets^5^, and void and gas bubble superlattices^6^, in pure metals, alloys, ceramics and semiconductors. Such patterned nano-structures have significant scientific merits and great technological importance for their novel physical properties. In particular, irradiation is a powerful tool to create far-from-equilibrium environments, which provides an opportunity to generate unique self-organizations, such as nanoscale compositional patterning in immiscible alloys^7^, patterning of defect clusters and loops, and void and gas bubble superlattices in pure metals and alloys (see refs^8^ and^9^ for detailed reviews). With irradiation, a continuous external perturbation exerts on a system, which activates a number of internal interactions and reactions among various kinds of defects, e.g., vacancies, self interstitial atoms (SIAs), foreign elements, and their agglomerates, etc. As a consequence, the self-organization during such a complicated process is strongly influenced by the competing kinetics that drives the system towards equilibrium and that of external stimulation keeps the system at far-from-equilibrium. One typical example is the nanoscale compositional patterning formation by the competing kinetics between thermal diffusion and athermal ballistic mixing^7^. In general, the evolution of a system under irradiation is dominated by the interplay among non-equilibrium thermodynamics, stimulation and reaction kinetics, the theoretical description of which is a long-term challenging issue.

A void superlattice features a certain lattice symmetry, usually coherent with the matrix with rare exceptions^10^, and a characteristic length (lattice parameter) which varies with materials and irradiation conditions (e.g., temperature and dose rate)^9^. From theoretical point of view, the self-organization of void superlattices requires an intrinsic instability that leads to the appearance of a periodic inhomogeneous structure with both characteristic symmetry and length (i.e., superlattice symmetry and parameter). In the literature, different theoretical approaches have been proposed to explain these two characteristic properties. The appearance of a characteristic length has been explained by thermodynamic instability^11,12^ analogous to the spinodal decomposition in solid and liquid solutions^13^, dynamic instability in reaction-diffusion systems (e.g., Turing instability)^14–17^, and long-range interactions (e.g., void-void elastic interaction)^18^. In principle, a characteristic length of of void distribution should emerge during phase separation (*i*.*e*., between a void *phase* and the matrix phase), provided the defect dynamics (including production, annihilation and reaction) is considered correctly. In Imada’s model, only constant defect production is considered, so that the critical coupling effect of defects through annihilation and reaction was lost^11^. In Veshchunov’s work, the phase separation analysis was done by assuming a quasi-stationary state^12^, and void superlattice was regarded as a consequence of spinodal decomposition of solid solutions in binary alloys. Therefore the theory cannot be applied to any unary systems. As a matter of fact, void superlattices have been widely observed in unary systems experimentally. The dynamic instability analysis in reaction-diffusion systems involves defect production, annihiliation and reactions, which captures the dynamic nature of defects, including SIAs, vacancies, their clusters and loops. However, it overlooks the thermodynamic origin of the void formation. A void is formed through the uphill diffusion and local accumulation of vacancies, which requires the description of the chemical potential gradient rather than the concentration gradient in conventional Fick’s law. Without an appropriate thermodynamic consideration, a complete understanding of the selection mechanism of defect microstructure cannot be achieved. In particular, the pattern selection by dynamic instability is very sensitive to the dynamic parameters especially near post-bifurcation regime, implying that distinctively different patterns may form in the same material system, which is inconsistent with experimental observations that the superlattice structure is unique in a given material. For voids in an elastically anisotropic matrix, the elastic interaction between voids could suggest a void distribution minimizing the total elastic strain energy at a given ratio of void radius *R* over superlattice parameter *a*_*L*_^18^. Theories along this line have been appealing as they can predict both the superlattice parameter and the symmetry. Recent 2D phase field simulations also demonstrate that indeed bubble superlattice can form in an elastically anisotropic matrix^19^. However, it has difficulties in explaining the long-range ordering at the early, nucleation stage^9^. Also it cannot explain the formation of void superlattices in body-centered cubic tungsten, which is elastically isotropic^8^. *As of today*, *a theory is yet to be developed that can couple thermodynamics and defect dynamics to successfully predict the experimentally observed superlattice parameters*. In addition to anisotropic elasticity, another mechanism proposed to understand the superlattice symmetry is anisotropic defect diffusion, such as 1D^20–22^ and 2D^23^ diffusion of self-interstitial atoms (SIAs) and/or SIA clusters/loops. These mechanisms, especially the 1D SIA and SIA cluster diffusion, seem consistent with many experimental observations, with support from recent 2D phase field^24,25^ and 3D objective kinetic Monte Carlo (KMC) simulations^26,27^. Also, recent atomistic calculations have shown that 1D diffusion is indeed the case for SIA in many body-centered-cubic (bcc) metals^28^, SIA cluster in bcc iron^29^ and face-centered-cubic (fcc) Ni^30^. *However*, *atomic scale perspectives on how SIA diffusion affects superlattice nucleation are yet to be discerned*.

This work focuses on the above two open issues. For the first time, the nucleation process of void superlattice is observed via atomistic simulations. Void superlattices form via spontaneous separation of a void *phase* from the matrix, analogous to phase separation in immiscible regular solid solution. The superlattice symmetry dictated by kinetic anisotropy such as 1D SIA diffusion. The phase separation is driven by thermodynamics and influenced by defect dynamics. Corresponding theoretical analysis leads to an quantitative prediction of superlattice parameter based on materials properties and irradiation conditions, without any fitting parameters. The unprecedented predictivity is demonstrated using independent experiments in bcc Molybdenum (Mo) and tungsten (W). The theory is also capable to guide new experiments in various materials and under different irradiation conditions.

## Methodology

### Thermodynamic and kinetic formulations

Our theory couples the rate theory for defect accumulation^31^ and the Cahn-Hillard approach for phase separation^13^. The evolution of time- and spatially-dependent concentrations, *c*_*v*_ and *c*_*i*_ for vacancy and SIA, respectively, are given by:

1 $$\frac{\partial {c}_{v}}{\partial t}=P(1-{c}_{v})+\nabla \cdot {M}_{v}\nabla (\frac{\delta F}{\delta {c}_{v}})-{k}_{iv}{c}_{i}{c}_{v}-{k}_{vs}{D}_{v}{c}_{v}$$

2 $$\frac{\partial {c}_{i}}{\partial t}=P(1-{c}_{v})+\nabla \cdot {D}_{i}\nabla {c}_{i}-{k}_{iv}{c}_{i}{c}_{v}-{k}_{is}{D}_{i}{c}_{i}$$

here subscripts *i*, *v*, *s* denote SIA, vacancy and sink, respectively. *P* is the production rate (or dose rate). The term (1 − *c*_*v*_) ensures mass conservation considering volumetric swelling. *M* and *D* denote atomic mobility and diffusivity, with the subscripts *i* and *v* for SIA and vacancy, respectively; *M* = *D*/*K*_*B*_*T*, with *K*_*B*_ being the Boltzmann constant. *F* is the total free energy of the system. *k*_*iv*_ is the reaction rate for recombination, while *k*_*vs*_ and *k*_*is*_ are those for sink absorption. And *k*_*iv*_ = 4*πR*_*iv*_(*D*_*i*_ + *D*_*v*_)/Ω; here *R*_*iv*_ is the instantaneous recombination radius and Ω is the atomic volume. Using *Q* = *k*_*iv*_*c*_*i*_ + *k*_*vs*_*D*_*v*_ + *P*, the vacancy evolution equation in eq. 1 can be reduced to:

3 $$\frac{\partial {c}_{v}}{\partial t}=P+\nabla \cdot {M}_{v}\nabla (\frac{\delta F}{\delta {c}_{v}})-Q{c}_{v}$$

with this mathematical form the theory is now generalized to phase separation with the source (*P*) and the reaction (*Qc*_*v*_) terms. The use of a free energy description in the spatially dependent diffusion term allows for the formation and migration of voids driven by the free energy. Effectively, voids are represented by local concentration c_*v*_ = 1, and they can precipitate out as a void *phase* from the matrix phase, in a way similar to phase separation in immiscible regular solid solution. Such an approach has been widely used for void formation under irradiation using the phase field method^24^. Note that the reaction term can be nonlinear since both *c*_*i*_ and *k*_*vs*_ in *Q* are coupled with *c*_*v*_. The theoretical formulation provides a phenomenological description of defects evolutions during void superlattice formation at the continuum level. It will be utilized later to predict the superlattice parameter. The above equation includes three indispensable pieces: (i) thermodynamics driving vacancy evolution, (ii) defect dynamics including production and annihilation, and (iii) non-linear coupling between opposite types of defects through recombination. Note that in existing theories usually only one or two of the three critical pieces were considered. For example, (ii) and (iii) are considered in the dynamic instability analysis^8,9^, while (i) and (ii) are considered in spinodal decomposition analysis^11,19^.

In analogy to the binary regular solution formulation, the total free energy of the system can be written as a function of *c*_*v*_ as:

4 $$F={\int }_{V}\,(f+\frac{1}{2}\kappa |\nabla {c}_{v}{|}^{2})dV$$

here *f* is the bulk free energy density of the *binary* mixture of vacancies and matrix metal atoms, given by

5 $$f={E}_{mix}{c}_{v}(1-{c}_{v})+{K}_{B}T({c}_{v}\,\mathrm{ln}\,{c}_{v}+(1-{c}_{v})\,{ln}\,(1-{c}_{v}))$$

*E*_*mix*_ is the heat of mixing (vacancy formation energy ${E}_{v}^{f}$ here). *κ* is the coefficient of gradient energy and it is associated with the interfacial energy *γ* (interface between void and matrix) approximately by $\kappa \cong 9{\gamma }^{2}\mathrm{/8}{E}_{mix}$^32^.

### Atomic kinetic Monte Carlo modeling and simulations

In accordance to the above theoretical framework, a rigid-lattice AKMC model for regular solution that incorporates 1D SIA diffusion for kinetic anisotropy is developed to explore the nucleation of void superlattice. Here, vacancy and SIA are denoted as types of elements occupying and diffusing on a prescribed lattice. Vacancies diffuse isotropically via first nearest neighbor (1NN) hopping, *i.e.*, switching with a matrix atom. To represent 1D SIA diffusion, multiple types of SIAs are used, each diffusing along a prescribed direction. Taking 〈111〉 1D SIA diffusion in bcc metals as an example, four types of SIAs are used, each diffusing along one of the four 〈111〉 directions by performing 1NN hopping. The 1D SIA diffusion can be turned off for 3D isotropic diffusion. Moreover, preferential 1D diffusion can be simulated by allowing one type of SIA to transform into another type with a given barrier. In this work, simulations are mostly carried out using pure 1D diffusion for the efficiency. The major conclusion holds as long as 1D diffusion is dominant, in agreement with previous work^26^. Following the residence-time algorithm^33^, in each AKMC step a list of diffusing events is built based on the jumping rate of each event *i*, *τ*_*i*_ = *ν*_0_ *exp*(−${E}_{a}^{i}/{K}_{B}T$); *ν*_0_ is the attempt rate and ${E}_{a}^{i}$ the activation barrier. A random number is drawn to select one event from the list to proceed in each KMC step. The time advancement is given by the inverse of the summation of all jump rates. A constant *ν*_0_ of 1.0 × 10^12^/s is used to scale the AKMC time to physical time. The activation barrier for vacancy diffusion is calculated by: *E*_*a*_ = *E*_0_ + (*E*_*f*_ − *E*_*i*_)/2, and updated once the local environment is changed. Here *E*_0_ is the diffusing barrier at the dilute concentration regime, and *E*_*f*_ − *E*_*i*_ is the energetic difference of the final and the initial states, describing the dependence on local environment. A constant activation barrier *E*_0_ is used for SIA for its low concentration at the condition for superlattice formation and its low migration barrier in the materials considered here. The total energy of the system is calculated by a pairwise model:

6 $$E=\frac{1}{2}\,\sum _{\alpha }\,\sum _{i}\,\sum _{j,j\ne i}\,{\varepsilon }_{\alpha }^{{e}_{i}{e}_{j}}$$

here ${\varepsilon }_{\alpha }^{{e}_{i}{e}_{j}}$ represents the bond energy between atom *i* (with the element type *e*_*i*_) and *j* (*e*_*j*_), within the *α*^*th*^ nearest neighbor shell, with *α* being 1 or 2 here. To sufficiently represent the free energy model in eq. 4, two terms in ${\varepsilon }_{\alpha }^{{e}_{i}{e}_{j}}$ need to be non-zero. In the current model, only ${\varepsilon }_{\alpha }^{12}$ are defined, with *e*_*i*_/*e*_*j*_ equaling to 1 for the matrix and 2 for vacancy, respectively. *a*_0_ is the lattice constant. For a bcc lattice, the bond energy can be derived by using,

7 $${E}_{mix}=8{\varepsilon }_{1}^{12}+6{\varepsilon }_{2}^{12}$$

8 $$\kappa =({\varepsilon }_{1}^{12}+{\varepsilon }_{2}^{12}){{a}_{0}}^{2}$$

here *a*_0_ is the lattice parameter. After each KMC step, vacancies and SIAs located within a given distance (*R*_*iv*_) from each other recombine (*i*.*e*., both changed to matrix atoms) instantaneously. To capture sink absorption, a mean free jump *N*_*s*_ is used. Vacancies or SIAs that have jumped more than *N*_*s*_ times will be eliminated (changed to matrix atom), corresponding to a sink strength of ${k}^{2}=\frac{2dim}{{N}_{s}{r}_{0}^{2}}$^34^ with *r*_0_ being the distance of each atomic jump and *dim* being the dimension of diffusion. The same *N*_*s*_ applies to vacancy and SIA, assuming neutral sinks. To describe defect production, Frenkel pairs are introduced randomly by assigning two randomly selected atoms to be a vacancy and an SIA, respectively. One Frenkel pair are introduced per time span *t*_*fp*_, corresponding to a dose rate of *P* = (*t*_*fp*_*N*)^−1^, with *N* being the total number of atoms in the system. This way of introducing defects corresponds to the electron irradiation condition. The AKMC method is implemented in the SPPARKS code^35^. For visualization the Ovito software is used^36^. Because the simulations here involve various lattice types, domain sizes and temperatures, the detailed simulation setup is described along with the corresponding results. Periodic boundary condition (PBC) is used for all AKMC simulations in this work. Rigorous examination on the finite size effect has been carried out by reproducing the simulation results using cells with various sizes.

## Results

### Superlattice symmetry selection from AKMC simulations

**Figure 1:**
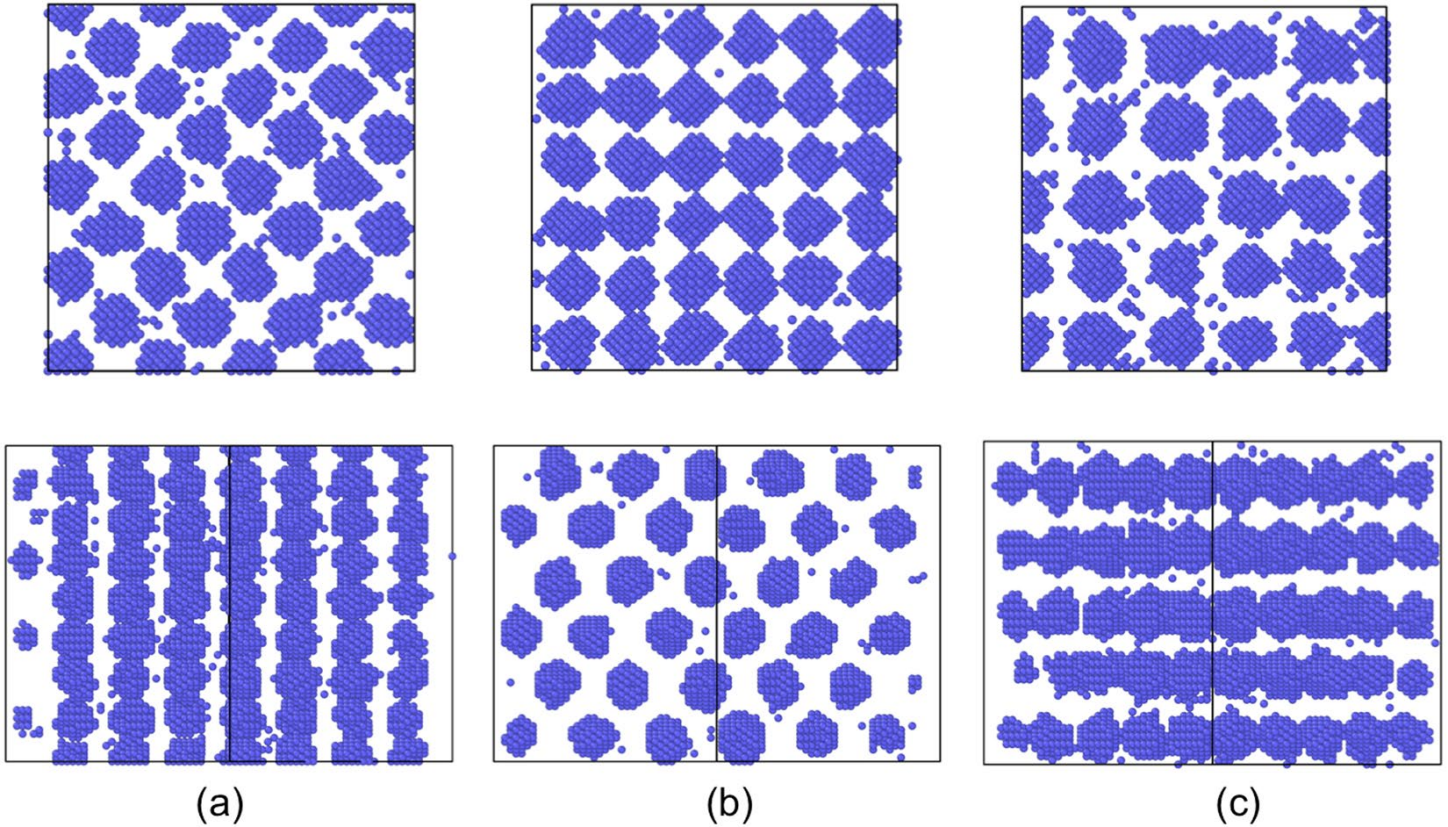
Snapshots from 3D AKMC simulations showing (**a**) bcc, (**b**) fcc and (**c**) simple cubic void superlattices formation. All simulations are performed with a bcc matrix with SIAs diffusing along (**a**) 〈100〉, (**b**) 〈110〉 and (**c**) 〈111〉 directions, respectively. Only vacancies are shown here. The simulation cell is cubic with all three axes along 〈100〉, with a dimension of 40 *a*_0_ × 40 *a*_0_ × 40 *a*_0_. Pictures on the top (bottom) are projected along 〈100〉 (〈110〉).

To explore how 1D SIA diffusion affects superlattice symmetry, the AKMC model is parameterized using the material properties of bcc Mo in Table 1. *R*_*iv*_ is set to be the 3rd nearest neighbor distance, and *N*_*s*_ be 1000. Both 2D and 3D simulations are carried out. For 2D, 1D SIAs diffusion along 〈10〉 is considered for square (sq) matrix, and along 〈10〉 and 〈11〉 for hexagonal (hex) matrix. The simulation cell size is 200 *a*_0_ by 200 *a*_0_ with 40000 atoms for square, and 200 *a*_0_ by 120 *a*_0_ with 48000 atoms for hexagonal matrix, respectively. For 3D, bcc and fcc matrices are used. For bcc matrix, 1D SIA diffusion along 〈100〉/〈110〉/〈111〉 directions are considered in three separated simulations, respectively. We note that for 〈100〉 and 〈110〉 1D diffusion in bcc, 2nd and 3rd nearest neighbor hopping need to be involved. For fcc matrix, 1D SIA diffusion along 〈110〉 is considered. The simulation cell size is 40 *a*_0_ by 40 *a*_0_ by 40 *a*_0_ for both bcc and fcc matrix, with 128000 and 256000 atoms, respectively. For all 2D and 3D simulations, void superlattices have been obtained with proper choices of irradiation conditions. The obtained superlattices from simulations are summarized in Table 2, consistent with all previous experimental observations. The void alignment, or the most-closed-packed direction of voids is found to follow the direction of 1D SIA diffusion once a superlattice forms. For instance, the most-closed-packed direction of voids is 〈111〉 for 〈111〉 1D SIA diffusion in a bcc matrix, giving a bcc void superlattice in 3D. Similarly, fcc and simple cubic (sc) void superlattices are observed in 3D simulations with 1D SIA diffusion along 〈110〉 and 〈100〉 directions (see Fig. 1), and square and hexagonal superlattices in 2D simulations with square and hexagonal matrices, respectively.

**Table 1:** Materials properties and production rate used in theoretical analysis and AKMC modeling.

Property	${{\boldsymbol{E}}}_{{\boldsymbol{v}}}^{{\boldsymbol{f}}}$	*γ*	${{\boldsymbol{E}}}_{{\boldsymbol{v}}}^{{\boldsymbol{m}}}$	${{\boldsymbol{E}}}_{{\boldsymbol{i}}}^{{\boldsymbol{m}}}$	*R*_*iv*_	*k*_*vs*_	*a*_0_
Unit	eV	*J*/*m*^2^	eV	eV	nm	10^12^/*m*^2^	*Å*
Mo	2.9^28^	2.95^44^	1.45^28^	0.083^45^	2.0^39^	14.2	3.15^28^
W	3.8^28^	3.47^44^	1.85^28^	0.054^45^	2.0	14.2	3.16^28^

**Table 2:** Void superlattices observed from AKMC simulations with 1D SIA diffusion.

Matrix	bcc	bcc	bcc	fcc	2D sq	2D hex
1D SIA diffusion direction	〈111〉	〈110〉	〈100〉	〈110〉	〈10〉	[10] & 〈11〉
Predicted superlattice	bcc	fcc	sc	fcc	sq	hex
Experimental observation	bcc^9^	fcc^10^	NA	fcc^9^*	NA	NA

*In fcc SIA clusters rather than individual SIAs perform 1D diffusion.

The above results cover a wide range of matrices including fcc, bcc, and 2D square and hexagonal. It confirms that 1D SIA diffusion can cause void alignment along SIA diffusion direction. In 3D, such alignment can take places along several symmetrical crystal orientations, e.g., 〈111〉 in bcc, resulting in void superlattice formation. This finding is consistent with previous theories^21^ and previous simulations^24,26^. The superlattice symmetry is dictated by the direction of 1D SIA diffusion, against the widely accepted empirical rule of the coherency between the superlattice and host matrix crystal lattice. The matrix lattice structure does not directly determine the structure of void lattice, although it has indirect effect by affecting SIA diffusion direction. Experimentally, bcc void/bubble superlattices have been observed in various bcc metals including Mo, W, Nb, Fe and Ta^6,8,9^. In all these metals, SIA has been predicted to perform 1D diffusion along 〈111〉^28^ except for Fe, in which SIA diffuses in 3D but SIA clusters primarily perform 1D diffusion along 〈111〉^29^. In bcc U-7Mo fuel where 〈110〉 1D SIA diffusion was suggested^25^, fcc void superlattices have been reported^10^. In addition to experiments, the current AKMC results are also consistent with previous 3D objective KMC^26^ and 2D phase field^24,25^ simulations.

### Rate theory based instability analysis

The above AKMC simulations adopt production rates orders of magnitude higher than those in the previous experiments. The observed superlattice parameters are usually a few nanometers in simulations, about one order of magnitude lower than those reported experimentally in bcc Mo^9^. This discrepancy can be resolved by theoretical analysis on the effect of irradiation conditions. The analysis starts with the Fourier form of eq. 3:

9 $$\frac{\partial {\tilde{c}}_{v}}{\partial t}=P\delta (k)-({M}_{v}\,f^{\prime\prime} {k}^{2}+{M}_{v}\kappa {k}^{4}+Q){\tilde{c}}_{v}.$$

**Figure 2:**
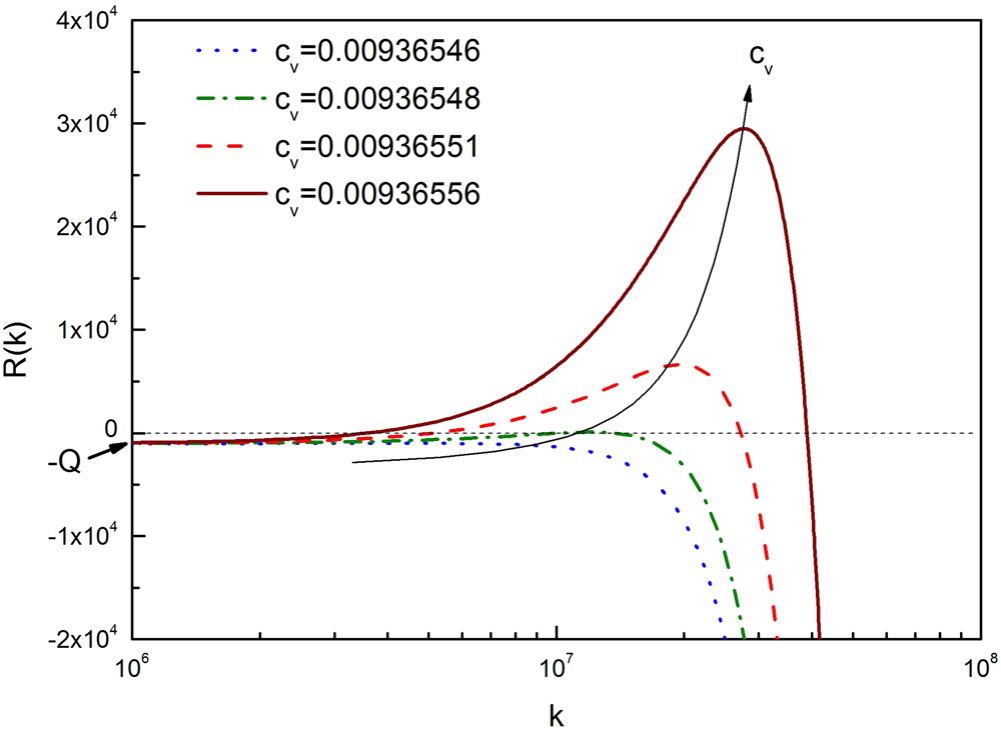
Growth rates of wave vectors at different vacancy concentrations with Q = 1000 *s*^−1^, E_*mix*_ = 3.03E10 *J*/*m*^3^, *κ* = 3.03E-10 *J*/*m*, and T = 1273 K.

In the Fourier space, the mean field concentration is described by the mode ${\tilde{c}}_{v}(k=\mathrm{0)}$, and spatial variations by non-zero *k* (*k* is the wave number). The production term is non-zero only for *k* = 0. Considering a small perturbation with a wave number *k*, its growth rate is given by $R(k)=-\,({M}_{v}\,f^{\prime\prime} {k}^{2}+{M}_{v}\kappa {k}^{4}+Q)$. For *Q* = 0, this reduces to spinodal decomposition in immiscible alloys^13^; when $f^{\prime\prime}  < 0$, there always exists non-zero *k* with positive growth rate *R*(*k*). When *Q* > 0 as in the case of irradiation or reaction, the spontaneous phase separation is delayed until $f^{\prime\prime} =-\,2\sqrt{\frac{\kappa Q}{{M}_{v}}}$, corresponding to a single and critical ${k}_{c}={(\frac{Q}{\kappa {M}_{v}})}^{\mathrm{1/4}}$ that satisfies *R*(*k*) = 0 and *dR*(*k*)/*dk* = 0, as shown by the green curve in Fig. 2. Accordingly, the critical concentration *c*_*v*_ can be calculated using eq. 5. The critical wave length is given by:

10 $${\lambda }_{c}=\frac{2\pi }{{k}_{c}}=2\pi {(\frac{\kappa {M}_{v}}{Q})}^{1/4}.$$

The appearance of *Q* in the denominator shows the strong coupling between phase separation and diffusion reaction (defect dynamics) in selecting void superlattice parameter. Therefore, it is critical to include both thermodynamics and defect dynamics in predicting superlattice parameter *a*_*L*_. Once the critical concentration is reached, slight increase in *c*_*v*_ leads to substantial increase in *k* and *R*(*k*), as shown in Fig. 2. The quick, exponential growth of the first *k* with positive *R*(*k*) will stabilize a characteristic length given approximately by eq. 10. This wave length corresponds to the inter-plane spacing of the most-closed-packed planes of voids, i.e., {110} for bcc crystals with 〈111〉 1D SIA diffusion, and thus ${\lambda }_{c}=\frac{\sqrt{2}{a}_{L}}{2}$.

### AKMC demonstration of instability

The theory predicts a spontaneous phase separation, *i*.*e*., separation of a void *phase* from the matrix, which determines the superlattice parameter depending on defect dynamics. This is consistent with the conclusion in Woo *et al*. that “From the view of thermodynamics, void-lattice formation is a non-equilibrium phase transition in an open system”^21^. As a support to the theory, AKMC simulations are performed to directly observe the superlattice nucleation and formation process and to investigate the dependence of superlattice parameter on radiation conditions such as temperature and dose rate. For these purposes, AKMC simulations are parameterized using the materials properties for both Mo and W as listed in Table 1. 1D SIA diffusion along 〈111〉 directions in a bcc lattice is considered. The simulation cells are 80 *a*_0_ by 80 *a*_0_ by 80 *a*_0_ in size with 1,024,000 atoms. Selected simulations have been repeated using 40 *a*_0_ by 40 *a*_0_ by 40 *a*_0_ and 120 *a*_0_ by 120 *a*_0_ by 120 *a*_0_ cells, with essentially the same results obtained on superlattice parameter and structure to exclude possible artificial effect from PBC. The dose rates are varied by two orders, being 0.98 and 98 dpa/s, respectively, to elucidate the dose rate effect. Because the AKMC simulation directly consider atomic hopping in both time and spatial scales, realistic dose rates as in real experiments are not achievable for the computation efficiency. The simulation temperature varies from 873 to 1473 K, one simulation every 100 K.

**Figure 3:**
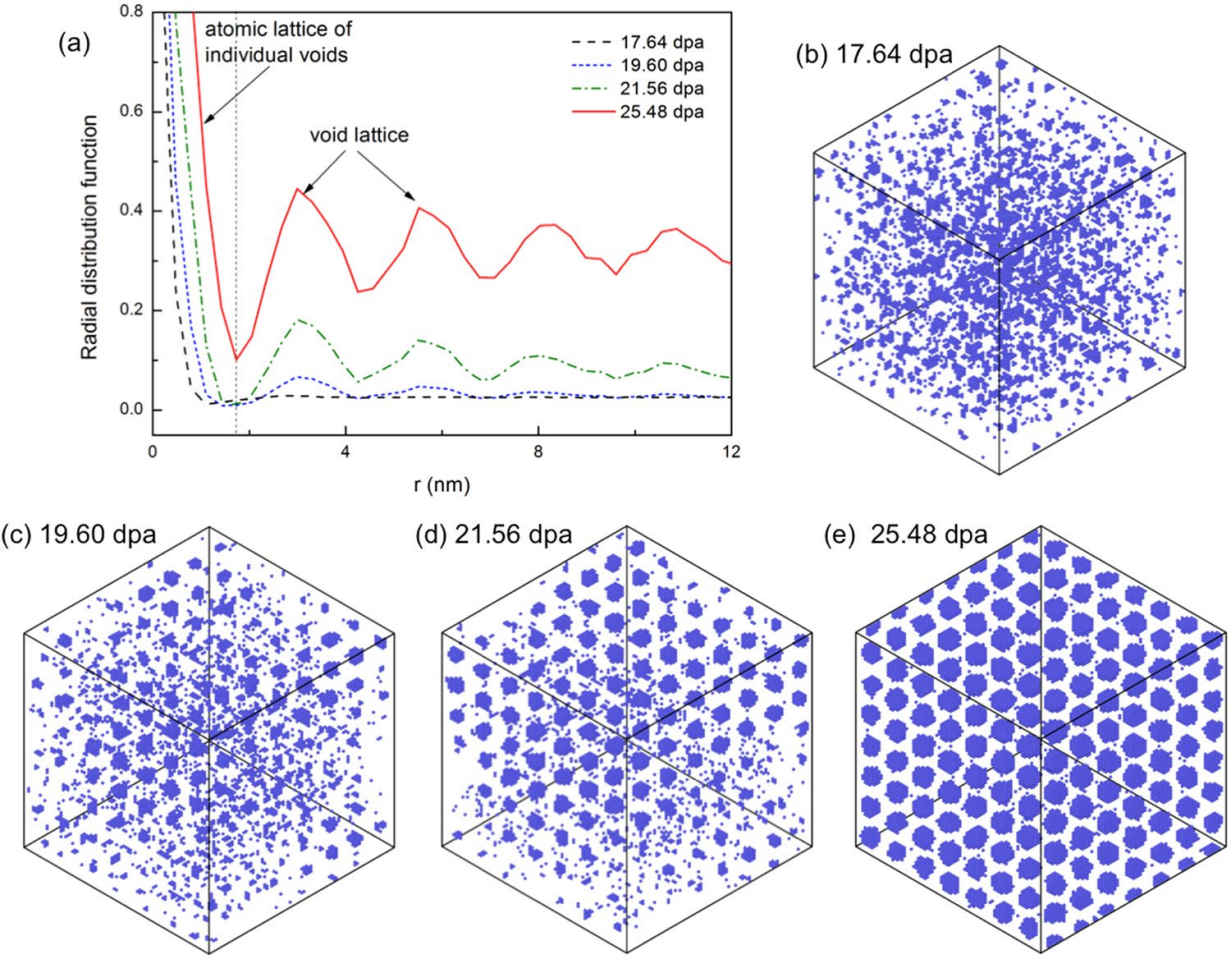
(**a**) Radial distribution function of vacancies at doses of 17.64, 19.60, 21.56 and 25.48 dpa. The corresponding atomic configurations are shown in (**b**–**e**), respectively. The simulation is done at 1173 K with a dose rate of 98 dpa/s. The simulation cell is cubic with all three axes along 〈100〉, with a dimension of 80 *a*_0_ × 80 *a*_0_ × 80 *a*_0_. The cell is tilted into 〈111〉 projection to show void alignment. A full movie of the simulation is available in the supplemental material 1173 K.mov.

To demonstrate the spontaneous separation of a void *phase* from the matrix, the atomic configurations at various doses from an AKMC simulation are plotted in Fig. 3, along with the radial distribution function of vacancies, *g*(*r*). The simulation is done at 1173 K with a dose rate of 98 dpa/s using the properties of Mo. Here *g*(*r*) is the number density of vacancies at the distance *r* from a vacancy, averaged over all vacancies in the system. As shown in Fig. 3(a,b), before the superlattice nucleates, only one peak in *g*(*r*) exists at short range, denoting the formation of individual voids. Once the critical condition for spontaneous phase separation is reached, extra and periodic peaks emerge at long range, indicating the appearance of a wave length (see Fig. 3(a)). The nucleation of a superlattice is clear in the corresponding atomic configuration in Fig. 3(c). Once a wave length is selected, its peaks grow quickly in amplitude without evolving in wave length, as shown in Fig. 3, which is typical for spontaneous phase separation. This indicates that voids are growing by absorbing mobile vacancies without coarsening, which is suppressed by the formation of a superlattice. Consequently, *a*_*L*_ is independent of dose in the AKMC simulation. Such a process has been observed in Tantalum (Ta)+ irradiated Mo at 900 °C^37^, where a static void superlattice parameter of 46.0 nm was observed from 3.0 to 150 dpa, while the void size has kept increasing. We note that coarsening can still occur when the void lattices contain imperfections.

**Figure 4:**
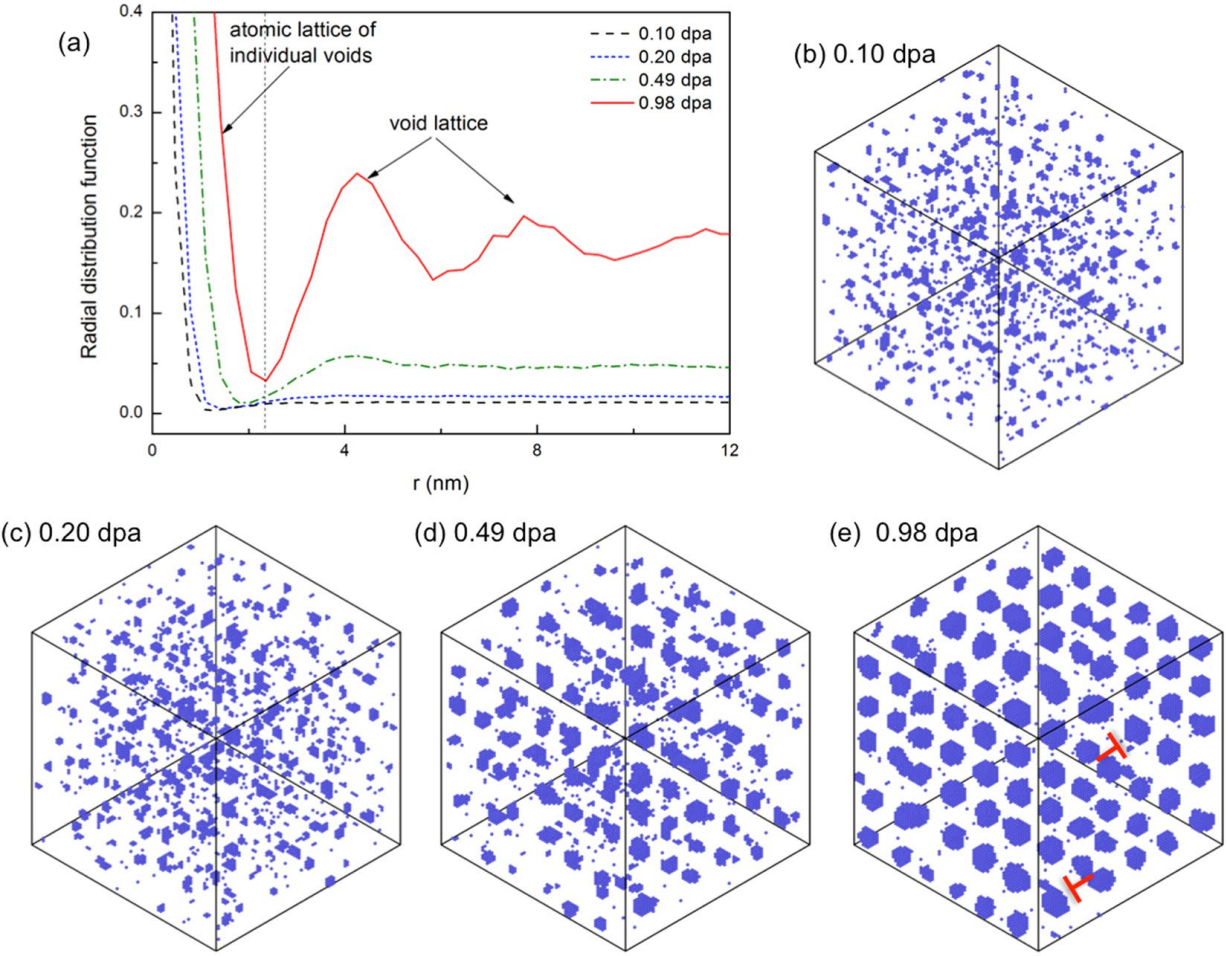
(**a**) Radial distribution function of vacancies at doses of 0.10, 0.20, 0.49 and 0.98 dpa. The corresponding atomic configurations are shown in (**b**–**e**), respectively. The simulation is done at 1373 K with a dose rate of 98 dpa/s. The simulation cell is cubic with all three axes along 〈100〉, with a dimension of 80 *a*_0_ × 80 *a*_0_ × 80 *a*_0_. The cell is tilted into 〈111〉 projection to show void alignment. A full movie of the simulation is available in the supplemental material 1373 K.mov.

The nucleation of void superlattice depends strongly on irradiation conditions such as temperature and dose rate. The effect of temperature can be observed in the simulation results at 1373 K and 98 dpa/s in bcc Mo. Compared to the case of 1173 K, at 1373 K there is not a clear nucleation stage, as shown in Fig. 4. In this case, individual voids form with weak alignment, as shown in Fig. 4(b,c). The alignment of voids improves as they grow larger, particularly after the critical condition for spontaneous phase separation has been reached, until a superlattice can be identified, Fig. 4(d,e). Accordingly, the *g*(*r*) curves (Fig. 4(a)) do not clearly reflect the selection and stabilization of a wave length, different from the case of 1173 K. The change in the formation mechanism with increasing temperature is due to the increased importance of individual void nucleation and growth, which is stochastic and leads to less ordering of the superlattice. As a result, the void superlattices usually contain imperfections, such as *vacant*, *sites* and *dislocations*, as shown in Fig. 4(e). These *defects* have been widely observed in previous experiments (See pictures in ref.^9^). It is expected that at even higher temperatures, individual void nucleation and growth become so dominant that no superlattice forms. Another notable effect is that at 1373 K, the dose needed for superlattice to form is substantially lower than that at 1173 K, due to the much stronger recombination at lower temperature. The reduction in dose rate has a similar effect as that exhibited by increasing temperature. Effectively, both of them drive the system *closer* towards equilibrium.

### Temperature and dose rate effects on wave length selection

The instability analysis above predicts strong dependence of void superlattice parameter on radiation conditions including temperature and dose rate. To validate that, eq. 10 is applied to bcc Mo and W, with the results compared to our AKMC simulations and previous experimental observations. To obtain *Q* it needs the transient SIA concentration and sink strength at the critical condition. Ideally, these can be obtained by solving the spatially dependent rate theory equations as used in the instability analysis^17^. A simpler estimate can be done by linearization of *Q*, *i*.*e*., solving eq. 1 assuming steady state and constant sink^31^ for an analytical solution of *c*_*i*_ (See Section I in the Supplemental Materials). The sink strength can be estimated based on the initial dislocation density and grain size in the samples.

**Figure 5:**
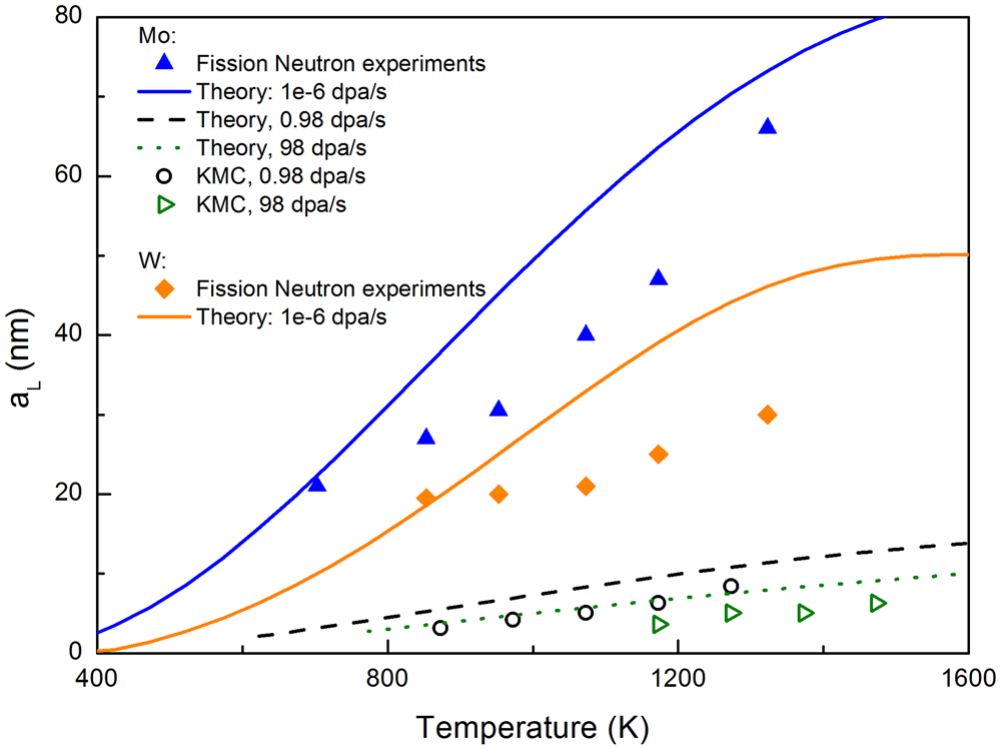
Lattice parameters of void superlattices as functions of temperature.

For comparison with experiments, the materials properties listed in Table 1 are used for Mo and W. Most of the parameters are from experiments except for *k*_*vs*_ and *R*_*iv*_. Here a value of 1.42 × 10^13^/*m*^2^ is used for *k*_*vs*_, corresponding to a dislocation density of 1.0 × 10^13^/*m*^2^ with a capture radius of 5 nm given by the Wiedersich model^38^. A dose rate of 10^−6^/*s* typical for fission neutron irradiation condition^9^ is used. The recombination radius *R*_*iv*_ has been found to be about 2.0 *a*_0_ for bcc Mo^39^. The same value is used for W. The same parameters are used for comparison with AKMC simulations except for the dose rates, recombination radius and sink strength. Two dose rates, 0.98 and 98 dpa/s, are used in AKMC simulations. The recombination radius is set as the third nearest neighbor distance, and the sink strength is given by *N*_*s*_ = 1.0 × 10^5^. The predicted superlattice parameters are plotted in Fig. 5 alongside the results from previous experiments and our AKMC simulations. Two sets of experiments^40^ are selected because they are done systematically to show the temperature effect with fixed dose and dose rate (the results are taken from ref.^9^).

At a given dose rate, for both W and Mo it is predicted that *a*_*L*_ initially increases with temperature due to increasing mobility, and then saturates due to increasing sink absorption. *a*_*L*_ is systematically larger in Mo than in W due to the higher vacancy mobility. These trends are consistent with experimental results from fission neutron irradiated Mo and W^40^. Notably, without using any fitting parameter in eq. 10, the predicted lattice parameters also agree well with experiments considering the uncertainties in the irradiation conditions and the materials properties. Such good comparison indicates that the theory captures the nature of superlattice formation and is capable for quantitative prediction. The discrepancies could be caused by several reasons including the uncertainty in materials properties and irradiation conditions. The initial dislocation densities are unknown in the experiments. The realistic vacancy mobility can be different due to the existence of impurities and the effect of irradiation enhanced diffusion. In fact, at low temperatures, irradiation enhanced diffusion in displacement cascades can be dominant over thermal diffusion. In such case, *a*_*L*_ will display a weak dependence on *T* as observed in the experiments for W.

The theoretical prediction are also in good agreement with AKMC results both qualitatively and quantitatively. *a*_*L*_ is predicted to increase with increasing *T* and decreasing *P*, as observed from AKMC simulations. At the same temperature, *a*_*L*_ observed from AKMC is larger with a dose rate of 0.98 dpa/s than with 98 dpa/s. As shown in Eq. 4, increasing in *P* will enhance defect recombination, resulting in increasing *Q* and thus smaller *a*_*L*_. The AKMC results are systematically below the theoretical curves. Two primary reasons are responsible for this minor discrepancy. First, a mean field distribution of individual vacancies is assumed in the theory. While in the simulation (and in reality) small vacancy clusters appear prior to the superlattice formation, as seen in Fig. 3. Thus the effective vacancy mobility in AKMC simulations is lower than that for individual vacancies, which is used in the theory, resulting in smaller *a*_*L*_ from the AKMC simulations than the from theoretical prediction. This effect becomes stronger with increasing temperature or decreasing dose rate when phase separation via void nucleation and growth becomes more important. The second is due to the periodic boundary condition, which allows for only discrete wave lengths. If the predicted wave length by eq. 10 is not compatible with PBC, phase separation will be delayed until a compatible *a*_*L*_ smaller than the theoretical one emerges. For this reason, the lattice parameters from periodic AKMC simulations should always be smaller than the theoretical prediction.

### Irradiation conditions to form superlattice

**Figure 6:**
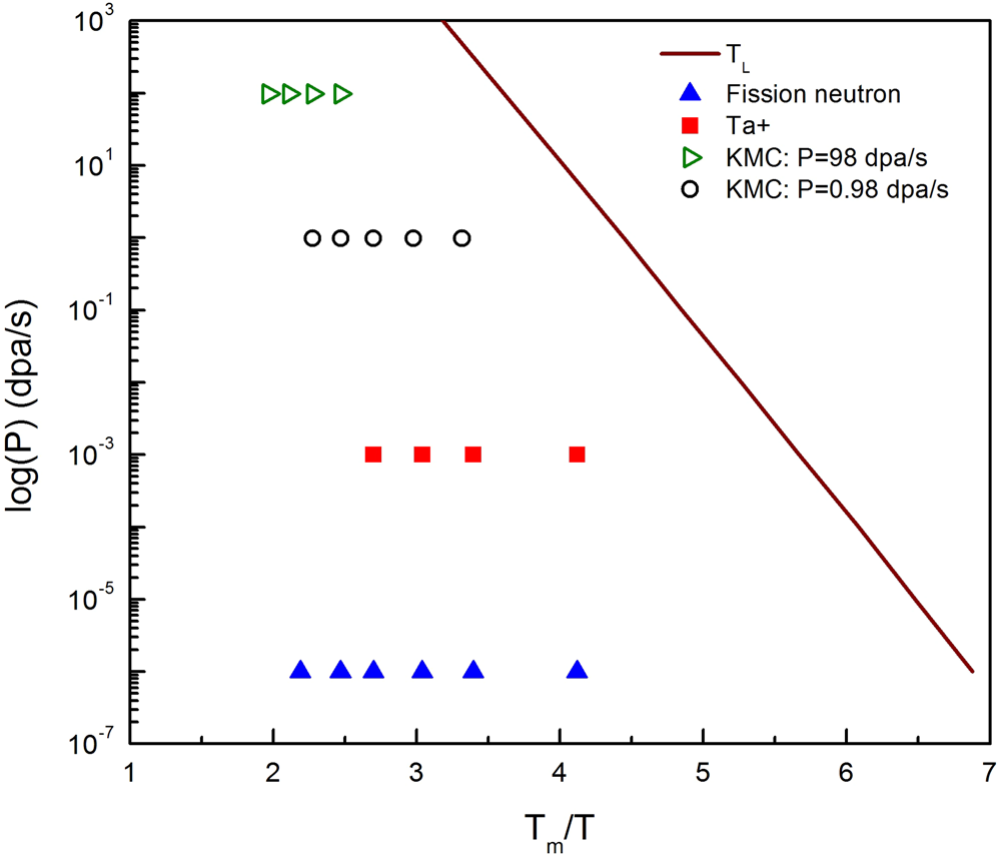
Temperature - flux diagram for superlattice formation for bcc Mo.

The theoretical analysis also predicts a low temperature boundary in the *P*–*T* diagram, which has been suggested previously by experimental data^9^. Following eqs 9 and 10, the low temperature boundary can be analytically solved for at the condition that no solution of *c*_*v*_ exists to satisfy *R*(*k*) > 0 for any *k*. More rigorously, the predicted distance between nearest voids, $\frac{\sqrt{3}{\lambda }_{c}}{\sqrt{2}}$, cannot be smaller than 2*R*_*iv*_, *i*.*e*., $2{R}_{iv} < \frac{2\sqrt{3}\pi }{\sqrt{2}}{(\frac{\kappa {M}_{v}}{Q})}^{\mathrm{1/4}}$. In such a condition, 1D SIA diffusion gives the same recombination as 3D diffusion, therefore there is no biased growth for aligned voids. This condition gives (See Section II in the Supplemental Materials):

11 $$P\leqslant \,\mathrm{ln}(\frac{9\kappa {\pi }^{4}{D}_{v0}}{4{R}_{iv}^{4}{K}_{B}{T}_{m}})+\,\mathrm{ln}\,\frac{{T}_{m}}{T}+\frac{-{E}_{mv}}{{k}_{B}{T}_{m}}\frac{{T}_{m}}{T}$$

here *D*_*v*0_ is the prefactor for diffusion and *T*_*m*_ the melting point. The thus established low *T* boundary is shown in Fig. 6, with all experimental and simulation conditions located at the higher temperature side. It also gives a nearly linear dependence of *ln*(*P*) on *T*_*m*_/*T*, with a slope of −${E}_{v}^{m}/({K}_{B}{T}_{m})$, as suggested in the literature^9^. The current analytical prediction does not suggest an exact high *T* boundary. In fact, what we see from AKMC simulations is that with increasing temperatures, voids become less ordered gradually due to void nucleation and growth (see Fig. 4), and coarsening becomes more active. In such cases, a superlattice may not be stabilized and identified. Another factor not considered here is the rotation of SIAs which breaks 1D diffusion. It is expected that, with increasing temperature, SIA diffusion will undergo a transition from 1D to 3D^29^, so that there is no long-range ordering of voids at high temperatures. For both the above reasons, superlattice formation gradually gives its way to stochastic void nucleation and growth, without a clear temperature boundary. These two effects could also play a role at temperatures with vacancy emission from voids^9^.

## Discussion

### Applicability of the theoretical model

The theory developed here contains no fitting parameters and is thus capable for quantitative prediction. The unprecedented predictivity is demonstrated by comparison to independent experiments and present AKMC simulations. Its analytical form makes it convenient to be applied for a wide range of materials and irradiation conditions including temperature and dose rate, as shown in Fig. 5. Three important trends regarding the superlattice parameter are predicted: i) *a*_*L*_ increases with increasing temperature, ii) *a*_*L*_ decreases with increasing dose rate, iii) under the same irradiation condition, *a*_*L*_ is larger in materials with higher vacancy diffusivities. The first and the third predictions are validated by the measurements in neutron irradiated Mo and W^40^, and the second one is validated by AKMC simulations. An indirect experimental support to the second prediction is that, in general, at the same temperature the void superlattice parameter produced by ion irradiation are usually smaller than that by neutron irradiation^9^, since the former is usually associated with much higher dose rates. Given the uncertainties in the experiments, quantitative comparison with experiments can be regarded as very good as well. The consistency between theory and experiments, alongside the direct support from AKMC simulations, indicates that the spontaneous phase separation based theory captures the nature of void superlattice formation. It can thus be utilized to tailor desired superlattice in experiments, e.g., by adjusting irradiation conditions and materials properties^30^.

The proposed theory is for void superlattice. It may be extended to explain gas bubble superlattice formation. One important effect of gas incorporation is that gas atoms occupy vacant sites, effectively reducing vacancy diffusivity. This will lead to much smaller superlattice parameter *a*_*L*_ according to eq. 10, consistent with previous experimental observations, where the bubble superlattice parameters are about one order of magnitude smaller than those for voids^9^. It is expected that bubble lattice will exhibit similar trends regarding temperature and dose rate. Unfortunately, so far no sufficient experimental data exists to establish the dependence of *a*_*L*_ on *P* and *T*. In our recent experiments of He implanted bcc Mo at 573 K, the bubble superlattice parameter was measured to be 4.0 nm at the dose rate of 1 × 10^−3^ dpa/s, and 4.8 nm at 1.2 × 10^−4^ dpa/s. Still, more data needs to be collected to establish a trend given the uncertainties in the experiments. Because of the high activation barriers for gas substitutes, irradiation enhanced diffusion becomes important for gas atom diffusion. Consequently, a weaker dependence of *a*_*L*_ on *T* is expected for bubble superlattices. These predictions are subjected to future validations.

### Limitation

Without the consideration of materials anisotropy, the thermodynamic instability itself does not predict a superlattice symmetry because in Eq. 10 only a wave length rather than a wave vector is predicted. In this work we rely on AKMC observations to predict superlattice symmetry. The results seem consistent with the “shadow effect” proposed in the literature^20^. It is showed that voids aligned along the 1D SIA diffusion directions receive lower annihilating SIA flux than unaligned ones^21^, resulting in superlattices in 3D. However, some AKMC simulations showed that such alignment may not be necessary during superlattice nucleation but appear after superlattice formation. This calls for further investigation on superlattice symmetry development. Actually, the absence of anisotropy in the matrix material makes the theory general for many materials, although it is demonstrated using primarily bcc metals in this work. In isotropic matrices, the voids will be randomly distributed, with the first nearest neighbor distance of *a*_*L*_ given by eq. 10. In such a case, voids are randomly distributed, and coarsening is expected to be active without identifiable void superlattices. In anisotropic matrices, ordering of voids can appear. In the case of 1D SIA diffusion when voids are aligned along the SIA diffusion directions, the stabilization of a characteristic length during spontaneous phase separation leads to superlattice formation, as shown in Fig. 3. The ordering will be weakened when phase separation via void nucleation and growth becomes dominant, as in the AKMC simulations shown in Fig. 4. We note that 1D SIA diffusion may not be the only factor for void ordering. For instance, the void ordering in bcc Fe and fcc metals are attributed to 1D SIA cluster (such as loops) diffusion^29,41^. 1D loop motion has been employed to explain void superlattice in bcc and fcc metals in general^22^. Other factors, like 2D SIA/SIA cluster diffusion^23^ and elastic anisotropy^18^, can also cause void ordering in certain ways. When elasticity is of concern, the present theory needs to be modified to include void elastic interaction in the free energy formulation^19^.

The present theoretical analysis assumes mean-field distribution of vacancies and SIAs before the critical point for spontaneous phase separation. In the rate theory description, vacancy and SIA types of defects are considered in the forms of concentration fields. We note that with spatial dependence and the free energy description, clusters of defects can also be represented as local variations in concentrations. For instance, a void can be represented by region with *c*_*v*_ = 1, taking the advantage that vacancy and voids are usually coherent with the matrix. They can be mobile driven by the total free energy as in Eq. 3, similar to classic phase field description^19,24^. Before the instability occurs, these clusters are unstable, *i*.*e*. waves with negative growth factors, and they may be annihilated by mutual recombination. Indeed, in the AKMC simulations, small vacancy clusters or voids are constantly observed (as shown in Fig. 4(b,c)) to form and vanish before superlattices nucleate. However, such an description may not be accurate for SIA clusters. Depending their size, SIA clusters may take various shapes and configurations. An accurate description requires distinction between them, as done in previous numerical approaches^17^ or the more complicated cluster dynamics description. This work focuses on the instability phenomenon in the vacancy concentration field. At the critical condition for instability, the SIA concentration is usually extremely low, e.g., below 10^−6^. With this condition, an simplification is made here by using the concentration variable *c*_*i*_ to describe SIA type of defects. For their role in recombining vacancies and absorbing SIAs, plus their possible anisotropic migration, their effects on superlattice parameter and symmetry^22^ warrant further investigations. Albeit the simplifications, we expect the present theoretical analysis to hold for various irradiation conditions, as indicated by the good agreement between the theoretical predictions and previous experiments.

The strong temperature dependence in the superlattice parameter is due to its direct correlation with vacancy mobility (diffusivity). There are other factors that may affect the temperature dependence, which are not included in the current theoretical analysis. In reality, both the recombination radius and the surface energy are temperature dependent. Irradiation enhanced diffusion in displacement cascades may weaken the temperature dependence, particularly under ion irradiation at low temperatures. In regards of these factors, the theory may not accurately reflect the temperature dependence of superlattice parameter observed in the experiments. Better agreement has been achieved between theory and AKMC, in both these factors are absent.

The last piece of discussion centers on the AKMC method used in this work. To demonstrate the instability phenomenon predicted by the rate theory, an AKMC method consistent with the rate theory description and capable of reach high radiation doses is needed. Moreover, prescribed SIA diffusion properties is desired to show the correlation between SIA diffusion and superlattice structure selection. For these purposes, we followed the AKMC method as described in^33^ by adding a description for anisotropic SIA diffusion. The AKMC simulations concern Frenkel pair production, corresponding to electronic irradiation. With this method, vacancy clusters and voids can form and migrate automatically via diffusion and clustering of individual vacancies. The clustering of SIAs is ignored for the extremely concentration at the condition for superlattice formation. The diffusion barrier of vacancy is calculated based on the local environment using a broken-bond model within the 2nd nearest neighbor distance. This model is sufficient to represent the two critical materials properties, surface energy and vacancy formation energy, to be consistent with the free energy description in the rate theory equations. Moreover, it provides the computation efficiency to reach high radiation dose with larger number of defects in the simulation domain. Therefore, it serves well the purpose of demonstrating the instability phenomenon predicted by the rate theory model. However, it may not be an ideal selection as a standalone tool for studying defect evolution for the simplified description of interatomic interaction and atomic diffusion. A more realistic description can be achieved by using an empirical potential for the interatomic interaction and advanced barrier searching methods for atomic diffusion^42,43^, in situations where computation efficiency is not a major concern.

## Conclusions

To conclude, atomic scale AKMC simulations confirm that void superlattice can form in various crystals with 1D SIA diffusion. The superlattice forms via spontaneous phase separation, with the characteristic length dictated by both vacancy thermodynamics and defect dynamics, and the lattice symmetry by 1D SIA diffusion. Assisted by the atomistic simulations, a new theory is developed to predict the superlattice parameter. The excellent agreements in both trends and exact magnitude between theory and independent experiments demonstrate that the theory is capable to interpret the mechanisms and make quantitative predictions, without using any fitting parameter. Further guidance on the experimental conditions for superlattice formation is also suggested by the our theory. The mathematical form of the theory implies that it may have general application in cases involving spontaneous phase transition and anisotropic diffusion reaction.

## Electronic supplementary material

1173K

1373K

Supplementary information
