# Supplementary material for: Theoretical prediction and atomic kinetic Monte Carlo simulations of void superlattice self-organization under irradiation
Source: Sci Rep. 2018 Apr 26;8:6629. doi: 10.1038/s41598-018-24754-9 (PMC5920090; doi:10.1038/s41598-018-24754-9)
Supplement: Supplementary file 3 — Supplementary information [file 41598_2018_24754_MOESM3_ESM.pdf]

Supplemental Information for:

Theoretical prediction and atomic kinetic Monte Carlo Simulations of void superlattice self-organization under irradiation

Yipeng Gao[1], Yongfeng Zhang[1], Daniel Schwen[1], Chao Jiang[1], Cheng Sun[2], Jian Gan[2], Xian-Ming Bai[3]

[1]*Fuels Modeling and Simulation, Idaho National Laboratory (INL), Idaho Falls, ID 83415, USA*
[2]*Advanced Characterization Department, Idaho National Laboratory (INL), Idaho Falls, ID 83415, USA*
[3]*Department of Materials Science and Engineering, Virginia Polytechnic Institute and State University, 460 Old Turner Street Blacksburg, VA 24061, USA*

I. DETERMINATION OF INTERSTITIAL CONCENTRATION

Our analysis in the main text focuses on the evolution of vacancy concentration described by eq. 3, in which Q is taken as a constant independent of time. However, in fact eqs. 1 and 2 are coupled together, and c_i is also included in Q. According to previous study in the literature, the concentration of interstitial can quickly approach its steady state concentration, and it is before the time point that the vacancy concentration reaches the level that allows void formation. As a result, it is reasonable to take the steady state concentration of interstitial to calculate Q.

The steady statue concentration can be determined for a homogeneous field, e.g., $c_i(r) = constant$. When the homogeneous system reaches steady state, we have

$$0 = \frac{\partial c_v}{\partial t} = P(1 - c_v) - k_{iv}c_ic_v - k_{vs}D_vc_v \tag{S1}$$

$$0 = \frac{\partial c_i}{\partial t} = P(1 - c_v) - k_{iv}c_ic_v - k_{is}D_ic_i \tag{S2}$$

By comparing eqs. S1 and S2, we have $k_{vs}D_vc_v = k_{is}D_ic_i$. Also consider $k_{vs} = k_{is}$, eq. S2 becomes,

$$0 = P(1 - \frac{D_i}{D_v}c_i) - k_{iv}c_i\frac{D_i}{D_v}c_i - k_{is}D_ic_i \tag{S3}$$

c_i at the steady state can be solved,

$$c_i^{st} = \frac{1}{2k_{iv}D_i}\left[\sqrt{(k_{vs}D_iD_v + PD_i)^2 + 4PD_ik_{iv}D_v} - k_{vs}D_iD_v - PD_i\right] \tag{S4}$$

And Q can be determined as,

$$\begin{aligned} Q =& k_{iv}c_i^{st} + k_{vs}D_v + P \\ =& \frac{1}{2D_i}\left[\sqrt{(k_{vs}D_iD_v + PD_i)^2 + 4PD_ik_{iv}D_v} - k_{vs}D_iD_v - PD_i\right] + k_{vs}D_v + P \end{aligned} \tag{S5}$$

Because all the kinetic coefficients, e.g., D_i and k_{iv}, depend on temperature, Q is a function of both temperature T and dose rate P.

II. NECESSARY CONDITION FOR VOID SUPERLATTICE FORMATION

From eq. 10, the critical wave length λ_c is determined by gradient coefficient κ, vacancy mobility M_v, sink strength Q (related to dose rate, emission rate and recombination rate). Note that λ_c does not depend on free energy density of the phase separate f explicitly. However, by solving the maximum value of $R(k)$, we have

$$f'' = -2\sqrt{\frac{\kappa Q}{M_v}} \tag{S6}$$

Note that the minimum of f'' is reached when $c_v = 0.5$,

$$f'' = -2E_{mix} + \frac{K_B T}{c_v(1-c_v)} \geqslant -2E_{mix} + 4K_B T \tag{S7}$$

So the necessary condition for the formation of void superlattice is,

$$-2\sqrt{\frac{\kappa Q}{M_v}} \geqslant -2E_{mix} + 4K_B T \tag{S8}$$

or

$$Q \leqslant \frac{M_v(E_{mix} - 2K_B T)^2}{\kappa} \tag{S9}$$

Note that another constraint, $\frac{\sqrt{3}\lambda_c}{\sqrt{2}} \geqslant 2R_{iv}$, should also be taken into account, so that the 1D SIA diffusion keeps its 1D nature even during recombination. Combined with eq. 10, we have

$$Q \leqslant \kappa M_v(\frac{9\pi}{4R_{iv}})^4 \tag{S10}$$

The above inequality (either eq. S9 or eq. S10) defines a region in P-T diagram, and the boundary of the region can be determined when equality is reached. Such a transcendental equation can be solved numerically. However, it can also be solved analytically by adapting a number of simplifications, without losing any key character. Note that since the equation defines the lower bound of temperature at given dose rate, the temperature is usually low, $\sim T_m/10$ (T_m is the melting temperature). As a result, all the diffusivity and mobility relating to temperature is relatively low, and one can expect,

$$k_{iv}c_i \ll P, k_{vs}D_v \ll P, K_B T \ll E_{mix} \tag{S11}$$

So eq. S9 and eq. S10 can be reduced when equality is reached, respectively,

$$P \approx Q \approx \frac{E_{mix}^2 M_v}{\kappa} = \frac{E_{mix}^2}{\kappa}\frac{D_{v0}}{K_B T}\exp(\frac{-E_{mv}}{K_B T}) \tag{S12}$$

$$P \approx Q = \kappa M_v(\frac{9\pi}{4R_{iv}})^4 = \kappa(\frac{9\pi}{4R_{iv}})^4\frac{D_{v0}}{K_B T}\exp(\frac{-E_{mv}}{K_B T}) \tag{S13}$$

Here we use the relation,

$$M_v = \frac{D_v}{K_B T} = \frac{D_{v0}}{K_B T}\exp(\frac{-E_{mv}}{K_B T}) \tag{S14}$$

D_v is diffusivity of vacancy, which can be determined by the maximal diffusion coefficient D_{v0} and the activation energy E_{mv} at a given temperature.

By using dimensionless parameter $t = T_m/T$, eq. S12 and eq. S13 can be simplified as,

$$P = \frac{E_{mix}^2}{\kappa}\frac{D_{v0}}{K_B T_m} t \exp(\frac{-E_{mv}}{K_B T_m} t) \tag{S15}$$

$$P = \kappa(\frac{9\pi}{4R_{iv}})^4 \frac{D_{v0}}{K_B T_m} t \exp(\frac{-E_{mv}}{K_B T_m} t) \tag{S16}$$

Two values of P can be obtained from the above equations. However, since we target on the upper bound of P, the smaller value of P should be our solution.

When $2E_{mix}R_{iv}^2 < 3\kappa\pi^2$, eq. S15 gives the solution. Making logarithm of eq. S15, we have

$$\ln P = \ln(\frac{E_{mix}^2 D_{v0}}{\kappa K_B T_m}) + \ln t + \frac{-E_{mv}}{K_B T_m} t \tag{S17}$$

So we get the function between $\ln P$ and t, and the slope of the curve is $1/t - E_{mv}/K_B T_m$. When $t \sim 10$, the first term is usually one or two magnitude smaller than the second one. So the curve deviates from linear slightly. As a matter of fact, it is suggested that there is a linear relation between $\ln P$ and t according to previous experimental observation and theoretical analysis.

When $2E_{mix}R_{iv}^2 > 3\kappa\pi^2$, eq. S16 gives the solution. And we have

$$\ln P = \ln(\frac{9\kappa\pi^4 D_{v0}}{4R_{iv}^4 K_B T_m}) + \ln t + \frac{-E_{mv}}{K_B T_m} t \tag{S18}$$

Note that according to the material parameters in Mo and W, $E_{mix}R_{iv}^2 > \kappa\pi^2$ is satisfied. So we use eq. S18 to determine the void superlattice region in P-T diagram (FIG. 6).
